# Association of alcohol intake with risk of diabetic retinopathy: a meta-analysis of observational studies

**DOI:** 10.1038/s41598-017-00034-w

**Published:** 2017-01-31

**Authors:** Wei Zhu, Yi-Fang Meng, Yan Wu, Ming Xu, Jiong Lu

**Affiliations:** 1 Department of Ophthalmology, Changshu NO. 2 People’s Hospital, Changshu, China; 2 Department of Ophthalmology, The First Affiliated Hospital of Soochow University, Suzhou, China

## Abstract

Diabetic retinopathy (DR) is a common microvascular complication of diabetes mellitus (DM). The associations of alcohol intake with DR risk have demonstrated contradictory results. Relevant studies were identified by searching electronic databases (Medline, EMBASE and Web of Science) until May 2016. We identified a total of 12,875 DR cases among 37,285 participants in 15 observational studies. The pooled estimation of all the included observational studies was 0.91 (95% CI, 0.79 to 1.06) in a random-effect model. Analyses stratified by study design showed no significant association between alcohol intake and DR incidence in cohort, case control or cross-sectional studies. In the subgroup analyses, neither beer nor spirits intake were associated with DR risk. Furthermore, it was interesting to find that protective effects were detected in the wine (OR = 0.77, 95% CI = 0.64 to 0.92) and sherry (OR = 0.22, 95% CI = 0.05 to 0.95) groups. In conclusion, this current meta-analysis demonstrated that alcohol intake was not associated with risk of DR. Subgroup analysis by alcoholic beverage types showed that wine consumption would reduce the incidence of DR. In the future, more large-scale prospective studies with detailed alcohol subtypes and contents are still warranted to clarify the association.

## Introduction

Diabetes mellitus (DM) is now regarded as one of the most challenging public health problems worldwide^1^. Both the economic burden and loss of health caused by complications of DM indicate the importance of primary prevention and early intervention in the management of DM^2, 3^. Diabetic retinopathy (DR) is a common microvascular complication of DM and a major preventable cause of visual impairment in the working-age population^4^. Accordingly, early detection of DR in the DM population is crucial. The mechanisms of DR development and progression are still not fully understood^5, 6^. Previous epidemiological studies indicated that longer DM duration, older age, aging and cardiovascular events were risk factors for DR incidence^7, 8^.

Alcohol intake is reported to be a risk factor for several kinds of diseases, including cancers, gastrointestinal diseases, respiratory disorders and infections^9–11^. A quantitative review of 26 epidemiological studies reported an inverse association between alcohol intake and risk of type 2 DM (T2DM) in a non-linear dose-response manner. It was found that light and moderate alcohol consumption was associated with a lower risk of T2DM, whereas heavy alcohol consumption demonstrated no protective effect against T2DM risk^12^. Furthermore, in an advanced study, specific types of alcoholic beverage consumption demonstrated modified influences on T2DM risk. In a meta-analysis of 13 prospective studies, compared with beer or spirits, wine was associated with a more significant inverse risk of DM^13^.

In previous studies, the associations of alcohol intake with DR risk demonstrated contradictory results. Alcohol intake, which was regarded as an unhealthy lifestyle behavior, was previously regarded as a risk factor for DR development^14^. Cardiovascular events, which were reported to be risk factors for DR, could be prevented by moderate alcohol intake. Moderate alcohol intake was thereby reported to be associated with a decreased risk of DR^15^. We therefore conducted a systematic review of all observational studies that reported a relationship between alcohol consumption and DR risk. To clarify the detailed effects of alcohol intake on the risk of DR, modified alcohol quantities and specific types of alcoholic beverages were collected. The purpose of this current study was to investigate the association between alcohol intake and DR risk by conducting a meta-analysis of observational studies such as cohort, case control and cross-sectional studies classified by type of study design.

## Materials and Methods

This current meta-analysis was conducted following Meta-analysis of Observational Studies in Epidemiology (MOOSE)^16^ and Preferred Reporting Items for Systematic reviews and Meta-Analysis (PRISMA) guidelines^17^.

### Search Strategy and Selection Criteria

A literature search was conducted by searching three electronic databases, including PubMed, EMBASE and Web of Science, for research published by May 2016. The search strategy combined the keywords and corresponding MeSH terms regarding alcohol intake (alcohol, drink, wine, beer and spirits) and diabetic retinopathy in observational epidemiological studies such as cohort, case control and cross-sectional studies. No restrictions were set in the literature search. Additionally, possible studies were detected by reviewing the references lists of the reviews or articles. When the data we needed was missing in the article, we attempted to contact the corresponding authors for additional information. All the literature searches were conducted by one author and then checked by another author.

The review included observational studies reporting the association between alcohol intake and DR risk. The studies were included when they met the inclusion criteria as follows: (1) consisted of cohort, case control or cross-sectional design; (2) reported the association of alcohol intake and DR risk; (3) presented relative risk (RR), odds ratio (OR) or original data that could lead to OR values. We excluded studies in which only DR progression, microvascular complications or visual impairments were reported in the outcome. If different literature reported the results from the same datasets, only the smallest reports were included in our meta-analysis. Additionally, the literature was excluded if no alcohol consumption data was detected.

### Data Extraction

Data extractions were conducted from each included study independently by two investigators (W Zhu and YF Meng). The following data were extracted by reviewing all the included studies: name of the first author, publication year, age and number of participants, study period, study site and design, DR designations and diagnostic methods. Additionally, the definition of alcohol intake, adjusting factor and the OR or RR with 95% CI were also recorded for advanced analyses. Any disagreements in the data extraction were resolved through discussion with the third reviewer until a consensus was reached.

### Quality Evaluation

Because there is no universal scale available for the evaluation of the quality of all kinds of observational studies, we developed a modified scoring system that was based on a commonly used system^18, 19^. The methodological quality of each included study was assessed by a checklist: (1) Defined study design (case control or cohort study, 1 point; cross-sectional study, 0 points), (2) List inclusion and exclusion criteria for all participants (Yes, 1 point; No, 0 points); (3) Indicate study period and follow-up duration (Yes, 1 point; No, 0 points); (4) Diagnosis of DR based on fundal examination or fundal photography (Yes, 1 point; No, 0 points); (5) Provided enrollment duration for all participants (Yes, 1 point; No, 0 points); (6) Described the general characteristics, such as age and sex, of the full participant population (Yes, 1 point; No, 0 points); (7) Adjusted for confounding factors, such as age, gender, DM duration or cardiovascular factors (Yes, 1 point; No, 0 points); (8) Stratified alcohol intake into more than three stratifications (Yes, 1 point; No, 0 points); (9) Common influence factors, including age, gender, DM duration, were matched among all the groups (Yes, 1 point; No, 0 points). Studies with over 6 points were considered to have relatively high methodological quality.

### Statistical Analysis

We evaluated the association between alcohol consumption and risk of DR by pooling the results from all the included studies. Adjusted OR or RR with 95% CI were used for the meta-analysis whenever possible. Crude values or primary data were used when adjusted data were not presented. Considering the existing heterogeneity among the modified observational study designs, we conducted this meta-analysis using the random-effects model in the presence of significant heterogeneity. In this study, both the I^2^ method and the χ^2^ test were used to detect heterogeneity. We assumed significant heterogeneity if I^2^ was greater than 50% or the P value was less than 0.1. We also conducted subgroup analyses by study design types (cohort studies, case control studies and cross-sectional studies). Furthermore, classified analyses of all the included studies were conducted by study sites, DR stage (PDR or any DR), DM type (T1DM or T2DM), alcohol consumption amount and consumption subtypes as well as adjusting status.

Sensitivity analyses were also conducted to evaluate the influence of individual studies on the final conclusion. A total of two methods were used in the sensitivity analysis: (1) drop the included studies individually and then calculate the modified effects; (2) conduct sensitive analyses through advanced analyses by cutting the studies with lower study quality. Potential publication bias was detected by both a funnel plot and Egger’s test. An asymmetric plot and P less than 0.05 demonstrate the possible existence of publication bias. When significant publication bias was detected, a trim and fill analysis^20^, which would yield the adjusted effect of funnel plot asymmetry, was conducted. All analyses were performed using STATA software (version 12.0; Stata Corp LP). P values less than 0.05 were considered statistically significant in each analysis.

## Results

### Literature Search

**Figure 1:**
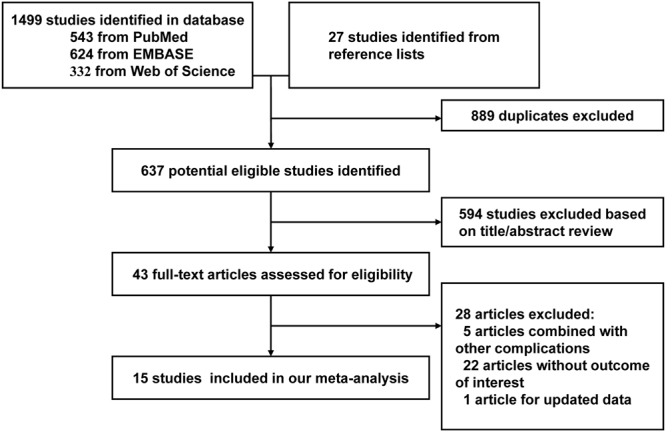
Flow diagram of the literature search and selection process.

Among 1499 publications retrieved by the electronic database search (543 from PubMed, 624 from EMBASE, 332 from Web of Science and 27 studies from reference lists), 889 duplicates were excluded. After reviewing title/abstracts of 637 articles, 594 studies were excluded after a first screening and 43 remaining articles were obtained for full-text review. In the full-text assessment for final inclusion, 28 articles were excluded for the following reasons: (1) DR was combined with other complications in 5 studies; (2) 22 studies were excluded either because they did not report outcomes of interest or no usable data was presented; (3) one study was excluded for updated data from a duplicated study. Finally, a total of 15 observational studies were included in this meta-analysis. The flow diagram of the literature search process is presented in Fig. 1.

### Study Characteristics

Table 1 shows the characteristics of all the included studies in this analysis. Among these included studies were cohort studies (n = 5), case control studies (n = 4) and cross-sectional studies (n = 6). The locations where the studies were conducted were as follows: Europe (n = 6), America (n = 2), Asia (n = 6) and Australia (n = 1). When DM types were considered, three studies focused on T1DM cases, and eight studies analyzed DR cases in T2DM patients. The DR identification in most studies was based on the Early Treatment Diabetic Retinopathy Study (ETDRS) severity level in 7 studies, whereas grade classification (3 or 4 levels) was used in 4 studies. The range of enrollment periods for included studies in this study was 1977–2014. All studies reported at least one alcohol consumption level as the exposure of interest. When the alcohol exposure definition was considered, a total of 7 studies demonstrated stratified analysis into more than three categories. The adjusted or matched factors in the included studies were also reported in Table 1. In the 15 observational studies, we identified a total of 12,875 DR cases among 37,285 participants.

**Table 1:** Characteristics of Eligible Studies.

Author year	Country	Study design	Age, year	DR diagnosis	DR definication	No. of case/control	Diabetes	Adjustment/matched	Exposure Definition
Yang JY 2013	Korean	CS	≥19	Fundus examination	ETDRS	112/866	Mix	Age, gender, smoking status, regular exercise, BMI, Serum total cholesterol, Serum triglyceride, Serum HDL-cholesterol, Anti-lipid drug use	≥4 alcoholic drinks/week, <3 drinks/week
Xu L 2009	China	Cohort	≥40	Fundus photographs	NR	366/3775	General	BMI, high-density lipoprotein, low-density lipoprotein, arterial hypertension	Consumers, Non-consumers
Moss SE 1994	USA	Cohort	≥21	Fundus photographs	ETDRS	238/678	Mix	Age, sex, glycosylated, hemoglobin, retinopathy	Average loz/day increase
Lee CC 2010	Whole world	Cohort	55–81	Retinal photography	ETDRS	640/599	T2DM	age, sex, HbA1c, systolic blood pressure, duration of diabetes, BMI, cigarette smoking, ethnicity	0, drinks ⁄week 1–14, drinks ⁄week >14 drinks ⁄week
Kohner EM 1998	UK	CC	25–65	Retinal photography	ETDRS	1102/1862	T2DM	NR	None, occasional, regular, heavy
Hirai FE 2007	USA	CS	45.3 ± 9.9	Retinal photography	ETDRS	309/228	T1DM	NR	Alcohol/No alcohol
Harjutsalo V 2014	Finnish	CS	28.9–46.8	Retinal photography	NR	1191/2417	T1DM	None	Heavy drinker light drinker
Fenwick EK 2015	Australia	CS	≥18	Fundus photography	ETDRS	235/160	T2DM	Education, income, language spoken at home, country of birth, lipid-lowering medication, hypertension medication	None, moderate, high
Young RJ 1984	UK	Cohort	20–59	Fundoscopic	Four Grades	230/62	Mix	NR	≤10 measures/week, >10 measures/week
Beulens JW 2008	European	CS	15–60	Retinal photographs	Three grades	304/2946	T1DM	Age, sex, centre, duration of illness, systolic BP, physical activity, smoking, BMI, presence of cardiovascular disease and HbA	0 g/week, 0.0–4.9 g/week, 5.0–29.9 g/week, 30.0–69.9 g/week, 70.0–209.9 g/week, ≥210 g/week
Rasmidatta S 1998	Thailand	CC	60.5 ± 7.4	Fundoscopic examinations	Three grades	63/135	T2DM	Glycosylated hemoglobin (%), cholesterol (mg/dL), triglyceride (mg/dL), HDL (mg/dL), BP (mmHg)	Nondrinker, drinker, not regular drinker
Jongsareejit A 2013	Thailand	CS	59.5	Indirect ophthalmoscope	International scales^a^	214/719	type 2	Gender, age, diastolic BP, waist, total cholesterol HDL, ccular perfusion pressure	No, ever, current
Martín-Merino E 2016	UK	CC	NR	Computerized records	NR	7735/9395	T2DM	Sex; age at index date; diabetes duration; primary care practitioner visits; referrals and hospitalizations; smoking; alcohol consumption; first HbA1c; systolic blood pressure; glaucoma; cataracts, or lens extraction; high-density lipoprotein and triglycerides; and hypoglycaemic agents, including oral hypoglycaemic drugs and insulin	0–1 units/week 2–21 units/week 22–34 units/week ≥35 units/week
Giuffrè G 2004	Italy	CC	≥40	Fundus examination	ETDRS	45/86	Mix	NR	None 1–19 years 20 years or more
Tseng ST 2015	China	Cohort	58.9	Funduscopic	Three grades	91/482	T2DM	NR	Drinker no-drinker

NR: not reported; CC: case-control study; CS: cross-sectional study; T1DM: type 1 diabetes mellitus; T2DM: type 1 diabetes mellitus; HDL: high-density lipoprotein; BMI: body mass index; BP: blood pressure; ETDRS: Early Treatment Diabetic Retinopathy Study.

^a^International clinical diabetic retinopathy and diabetic macular edema disease severity scales.

### Quality Scale of Study Methodology

The methodology quality of studies included in this meta-analysis is presented in Table 1 and Supplemental Table 1. The study methodologies were scored on a 9-point scale. The range of methodological scores was 3 to 8 points, and the average score was 6.07 points. Relatively high quality (6 points or more) was detected in 12 of the 15 included studies.

### Alcohol Intake and DR Risk

**Figure 2:**
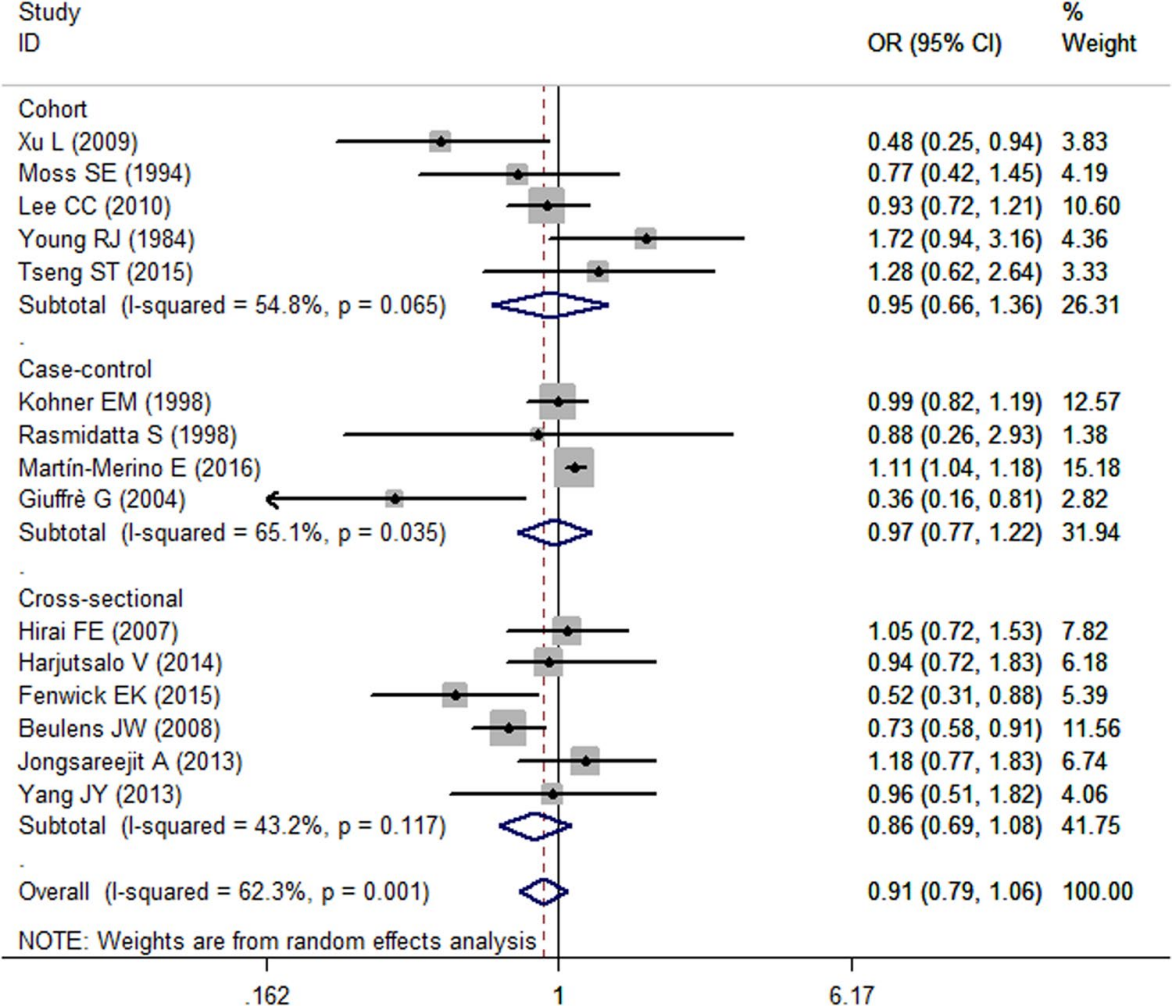
Summary of the odds ratios for the association between alcohol intake and the risk of DR by study designs. Through subgroup analyses, no significant association was detected in cohort, case-control or cross-sectional studies. The size of the shaded square is proportional to the percent weight of each study. Horizontal lines represent 95% CIs. The diamond data markers indicate pooled ORs.

The pooled estimation of the 15 included observational studies that reported the association between alcohol consumption and DR risk was 0.91 (95% CI, 0.79 to 1.06; Fig. 2) in a random-effect model. When heterogeneity was considered, significant heterogeneity was detected (*I* ^*2*^ = 62.3%, *P* = 0.001). Analyses stratified by study design showed that no significant association was evident between alcohol intake and DR incidence in cohort, case control or cross-sectional studies (cohort study: OR = 0.95, 95% CI = 0.66 to 1.36; case control: OR = 0.97, 95% CI = 0.77 to 1.22; cross-sectional study: OR = 0.86, 95% CI = 0.69 to 1.08).

In advanced subgroup studies, no significant associations were detected in Europe (OR = 0.94, 95% CI = 0.75 to 1.17), America (OR = 0.97, 95% CI = 0.70 to 1.34) or Asia (OR = 0.94, 95% CI = 0.66 to 1.33). However, only one study in Australia was included in this study, and an inverse association between alcohol intake and DR risk was detected (OR = 0.52, 95% CI = 0.31 to 0.88). When DM types were considered, no significant association was detected in either T1DM (OR = 0.84, 95% CI = 0.60 to 1.20) or T2DM (OR = 1.01, 95% CI = 0.87 to 1.16) group. Neither population-based (OR = 0.88, 95% CI = 0.74 to 1.04) nor hospital-based studies (OR = 1.17, 95% CI = 0.82 to 1.67) demonstrated significant association between alcohol consumption and DR risk. We also considered the alcohol amount intake and DR incidence. Through the stratified studies, neither protective nor harmful effects were detected among different alcohol consumption groups (light: OR = 1.10, 95% CI = 0.87 to 1.40; moderate: OR = 0.91, 95% CI = 0.72 to 1.14; heavy: OR = 0.90, 95% CI = 0.76 to 1.08). Otherwise, we measured the effects of different types of alcoholic beverages on the incidence of DR. In the subgroup analyses, intake of neither beer (OR = 0.90, 95% CI = 0.76 to 1.08) nor spirits (OR = 1.00, 95% CI = 0.81 to 1.24) was associated with DR risk. Furthermore, it was interesting to find that protective effects were detected in the wine (OR = 0.77, 95% CI = 0.64 to 0.92) and sherry (OR = 0.22, 95% CI = 0.05 to 0.95) groups. Further subgroup analyses on the adjusting status, age adjustment, gender adjustment and BMI adjustment showed no significant association in each subgroup. The detailed results are presented in Table 2.

**Table 2:** Subgroup Analysis of Alcohol Intake and Risk of DR.

	Subgroups	No. of studies	Summary Effect	P value	Study	Heterogeneity
			OR (95% CI)		I^2^, %	P value
DR stage	PDR	2	0.90 (0.55 to 1.45)	0.65	71.5	0.1
	Any DR	14	0.94 (0.80 to 1.09)	0.385	26.7	0.008
Study type	Cohort	5	0.95 (0.66 to 1.36)	0.612	54.8	0.065
	Case-control	4	0.97 (0.77 to 1.22)	0.807	65.1	0.035
	Cross-sectional	6	0.86 (0.69 to 1.08)	0.188	43.2	0.117
Site	Europe	6	0.94 (0.75 to 1.17)	0.506	77.9	<0.001
	Americas	2	0.97 (0.70 to 1.34)	0.843	0	0.414
	Asia	6	0.94 (0.66 to 1.33)	0.713	29.9	0.222
	Australia	1	0.52 (0.31 to 0.88)	0.015	—	—
DM type	Type 1	3	0.84 (0.60 to 1.20)	0.187	62.1	0.104
	Type 2	8	1.01 (0.87 to 1.16)	0.942	43.3	0.102
Alcohol amount	Light	3	1.10 (0.87 to 1.40)	0.43	78.3	0.003
	Moderate	6	0.91 (0.72 to 1.14)	0.397	71.2	0.004
	Heavy	8	1.06 (0.93 to 1.21)	0.412	16.8	0.001
Alcohol type	Beer	3	0.90 (0.76 to 1.08)	0.267	0	0.55
	Wine	3	0.77 (0.64 to 0.92)	0.005	0	0.472
	Spirit	3	1.00 (0.81 to 1.24)	0.975	0	0.545
	Sherry	1	0.22 (0.05 to 0.95)	0.042	—	—
Adjustment status	Adjusted	9	0.85 (0.69 to 1.05)	0.124	70.5	0.001
	Unadjusted	6	1.00 (0.77 to 1.30)	0.132	40.9	0.081
Age adjusted	Yes	6	0.95 (0.78 to 1.15)	0.584	65.8	0.012
	No	9	0.83 (0.62 to 1.10)	0.188	64.7	<0.001
Gender adjusted	Yes	6	0.95 (0.78 to 1.15)	0.584	65.8	0.012
	No	9	0.83 (0.62 to 1.10)	0.188	64.7	<0.001
BMI adjusted	Yes	4	0.79 (0.63 to 0.99)	0.038	34.5	0.205
	No	11	0.97 (0.82 to 1.15)	0.711	54.9	0.018

DM: diabetic mellitus; DR: diabetic retinopathy; OR: odds ratio; CI: confidence interval.

### Sensitivity Analyses and Publication Bias

**Figure 3:**
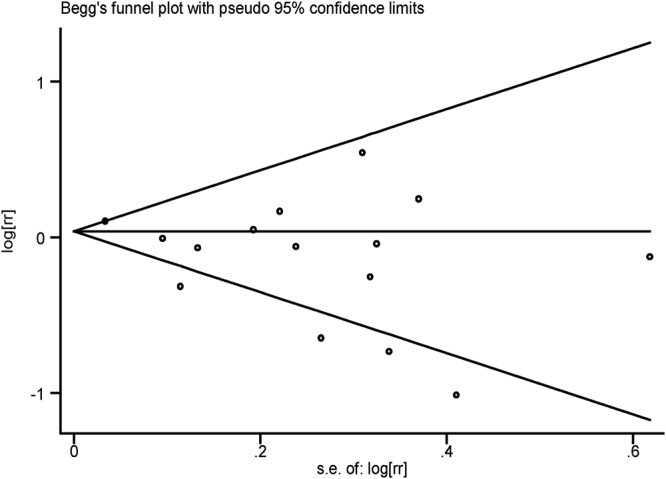
Funnel plot for the association between alcohol intake and the risk of DR in all the included studies. No significant publication bias was detected through pooling the 15 observational studies together.

**Figure 4:**
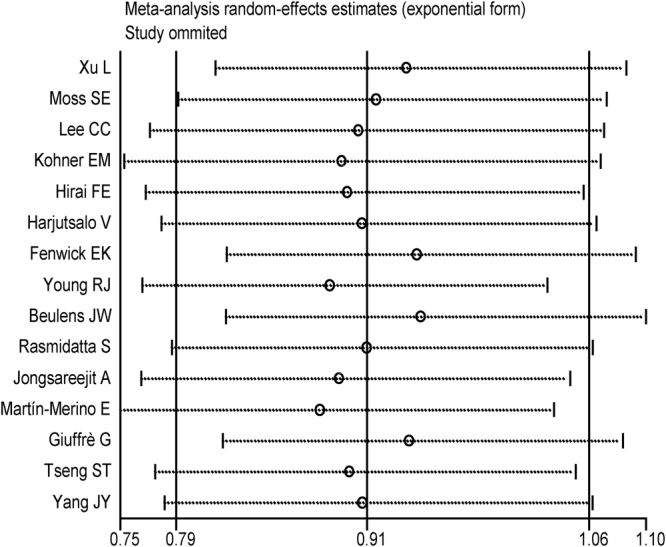
Sensitivity analysis through a one-way method. There were no studies influencing the result of alcohol consumption on DR.

In the sensitivity analysis, it was found that the outcome was not significantly changed when any study was excluded from this meta-analysis (Fig. 3). We also excluded studies with lower methodological scores and found no significant association between alcohol intake and DR risk (OR = 0.87, 95% CI, 0.73 to 1.04). Both visual inspection of Begg’s funnel plot and Egger’s test were used to detect publication bias. In general, it revealed symmetry for the funnel plot (P = 0.692, Fig. 4). However, Egger’s test demonstrated a significant publication bias (P = 0.049). Therefore, we conducted an advanced sensitivity analysis using the trim and fill method. It was found that no study should be added; thus, no advanced meta-analysis could be calculated with updated records.

## Discussion

There were contradictory results regarding the relationship between alcohol consumption and DR incidence in DM cases. This current meta-analysis showed that alcohol intake was not associated with DR risk in quantitative analyses of 15 observational studies. In advanced subgroup analysis, no significant associations were detected in different DR stages, study designs, DM type and various alcohol consumption groups. However, the subgroup meta-analyses by alcoholic beverage groups showed that wine and sherry intake were associated with reduced DR incidence. Overall, the sensitivity analysis indicated that the conclusions of this study were quite robust.

Alcohol consumption was regarded as a significant factor influencing increased morbidity and mortality^21^, while moderate alcohol intake demonstrated certain protective effects on cardiovascular diseases and DM status. A previous observational study with 5.5 years of follow-up indicated that light and moderate alcohol consumption could reduce the risk of T2DM^22^. In a dose-response meta-analysis of 38 observational studies, the results showed that significant reductions in the risk of T2DM were present at all levels of alcohol intake <63 g/day^23^. Considering that alcohol consumption was a protective factor of DM, it was natural to conjecture that alcoholic beverage intake might also influence the incidence or progression of the complications of DM. The incidence of DR, which was an important microvascular complication of DM, was influenced by various genetic and environmental factors. Considering that alcoholic beverages are usually consumed on a global scale, it is an important public health issue to determine the effect of alcohol consumption on the risk of DR.

It has been a long time since the associations between alcohol consumption and ocular disorders or visual health were first detected^24, 25^. In a previous cohort study, the relationships between alcohol patterns on the incidence of visual impairment (VI) in over a 20-year period were studied. Through analyzing the follow-up data, it was found that those who had not consumed alcoholic beverages over the past year had higher odds of incident VI than persons who drank occasionally^26^. The effect of alcohol intake on the VI incidence of DM patients was also detected in the Sankara Nethralaya Diabetic Retinopathy Epidemiology and Molecular Genetics Study (SN-DREAMS). Compared with that of non-drinkers, alcohol consumers demonstrated a lower prevalence of DR (OR = 0.41; 95% CI, 0.17 to 0.96; P = 0.04)^27^. The effect of alcohol consumption on DR risk was studied in previous epidemiological studies. As early as 1984, the role of alcohol consumption on development and progress of retinopathy was sought in 296 randomly selected diabetic men aged 20–59 during a five-year prospective study^28^. The results of the follow-up demonstrated that alcohol intake was associated with development and progression of retinopathy (P = 0.02). A cohort analysis of 1239 participants with T2DM aged 55 to 81 years enrolled in the AdRem study showed that moderate alcohol consumption, compared with no current alcohol intake, was not associated with presence or progression of diabetic retinopathy after adjusting for confounding factors^29^. Furthermore, in this cross-sectional study, the effects of the consumption of different alcoholic beverages on the risk of DR were reported among patients with T2DM^15^. Compared with the no alcohol intake group, moderate alcohol consumption was significantly associated with a reduced odds ratio of DR incidence (OR = 0.47, 95% CI, 0.26 to 0.85), while heavy alcohol consumption was not associated with DR risk (OR = 0.75, 95% CI, 0.25 to 2.20). In this study, through meta-analysis of 15 observational studies, it was reported that alcohol consumption was not associated with DR risk. Sensitivity analysis further indicated that the conclusions of this study were quite robust.

In the subgroup analysis by alcoholic drink types, wine and sherry consumption were associated with reduced risk of DR. This was an interesting finding with several potential explanations. Considering the relatively higher quality of wine and sherry compared with beer and spirits, the modification of drinking patterns of different alcoholic drink types might influence the DR risk. Drinking patterns might also be associated with healthy dietary behaviors and thus might affect the incidence of diabetes complications. Additionally, the polyphenolic content was the principal biologically active component in wine and was notably higher in fortified wine. Polyphenolic content could produce significant protective effects on DR progression both by reducing oxidative stress and inflammation in the retina directly and by decreasing cardiovascular and renal complications, thus reducing DR risk. However, the reason why no significant protective effect was detected from red wine as in a previous study is still unknown. Further epidemiological and *in vitro* studies will be required in the future. Considering that only limited studies have demonstrated the alcoholic drink subgroup effect on DR incidence (3 studies for wine and 1 for sherry), more well-designed studies with stratified analyses by alcohol types will be required in the future.

As this is the first meta-analysis on the association between alcohol consumption and DR risk as we know to date, a protective effect of wine consumption was detected. There are several possible mechanisms explaining this outcome. Moderate alcohol intake, especially of wine, has been regarded as a protective factor for cardiovascular disease^30^. In most epidemiological studies, a significant U-shaped association between alcohol consumption and all-cause mortality and vascular deaths was detected^31^. Through the SUN Project with 142,177 person-years of follow-up, the traditional Mediterranean alcohol-drinking pattern was reported to be associated with lower cardiovascular risk in most point estimates^32^. As cardiovascular events have been regarded as risk factors for DR^33^, the benefit of wine intake might arise from the reduction of cardiovascular disease risk. Furthermore, abnormal inflammation and increased oxidative stress have been regarded as key factors in the pathogenesis of DR development^34^. Both *in vitro* and *in vivo* studies have shown that polyphenol, delphinidin and resveratrol from wine produce protective effects against the development of DR through inhibiting the inflammatory response and oxidative stress^35^ ^,36^. Increased vascular endothelial growth factor (VEGF) activity also promoted the incidence and progression of microvascular complications of DM cases^37^. Moderate alcohol, especially red wine intake, demonstrated reduced VEGF expression^38^ and thus could produce a protective effect against DR risk.

The main strength of this meta-analysis was the comprehensive literature search. It was based on an extensive search up to May 2016 for potential included studies to identify associations between alcohol consumption and DR risk. In this study, the publication date of all included studies ranged from 1984 to 2015. Furthermore, the inclusion criteria in this study contained all observational studies reporting the effects of alcohol intake on the risk of DR. Comprehensive inclusion of all published studies provided stronger evidence for the conclusion. A stratified analysis by different subgroups would demonstrate a more detailed understanding of the effect of alcohol intake and risk of DR. Advanced studies by subgroup analysis showed that alcoholic beverage subtypes might produce differential effects on DR risk. The analysis provided clues for subsequent clinical study design and indicated that effects of alcoholic beverage types should be comprehensively explored in studies with more participants. Detailed information on the frequency and type of alcoholic drinks as well as other dietary or social factors should be collected and analyzed. However, certain limitations existed in our current meta-analysis that also must be considered. First, although 15 observational studies were included in this study, six of them consisted of cross-sectional design. The cross-sectional design is considered to demonstrate a weaker power to detect correlations. The relatively lower number of cohort studies included in this analysis demonstrated the requirement for additional well-designed cohort studies. Second, publication bias, which might influence the robustness of the conclusion, was detected in this study. The P value in Egger’s test was 0.049, and no additional studies were indicated using the trim and fill method. However, only mild significance was presented in the publication bias calculation, and the unmodified result was detected through trim and fill analysis. Thus, publication bias did not have a crucial influence on the conclusion of this study. Third, the crude OR/RR was provided in several included studies. Additionally, the adjusted models were different among the included studies. Accordingly, adjusting status might play an important role in DR development.

In conclusion, this current meta-analysis demonstrated that alcohol intake was not associated with the risk of DR. Subgroup analysis by alcoholic beverage type showed that wine consumption could reduce the incidence of DR. No significant association was detected between different quantities of alcohol intake and DR incidence in our meta-analysis. In the future, more large-scale prospective studies with detailed alcohol subtypes and contents are still warranted to clarify the association.

## Electronic supplementary material

Supplementary Information
