# Supplementary material for: Association of alcohol intake with risk of diabetic retinopathy: a meta-analysis of observational studies
Source: Sci Rep. 2017 Jan 31;7:4. doi: 10.1038/s41598-017-00034-w (PMC5428369; doi:10.1038/s41598-017-00034-w)
Supplement: Supplementary file 1 — Supplementary Information [file 41598_2017_34_MOESM1_ESM.pdf]

Association of alcohol intake with risk of diabetic retinopathy: a meta-analysis of observational studies

Wei Zhu [1, †], Yi-Fang Meng [1, †], Yan Wu [2], Ming Xu [1], and Jiong Lu [1,*]

Supplemental Table 1. Quality Scales of Study Methodology

Author, year	A	B	C	D	E	F	G	H	I	Total
Yang JY, 2013	0	1	0	1	1	1	1	0	1	6
Xu L, 2009	1	1	1	1	1	1	1	0	0	7
Moss SE,1994	1	1	1	1	1	1	1	0	1	8
Lee CC, 2010	1	1	1	1	0	1	1	1	1	8
Kohner EM, 1998	1	1	0	1	1	1	0	1	0	6
Hirai FE, 2007	0	1	0	1	1	1	0	0	0	4
Harjutsalo V, 2014	0	1	0	1	0	1	0	0	0	3
Fenwick EK, 2015	0	1	0	1	1	1	1	1	0	6
Young RJ, 1984	1	1	1	1	1	1	0	0	0	6
Beulens JW,2008	0	1	0	1	1	1	1	1	1	7
Rasmidatta S,1998	1	1	0	1	1	1	0	1	0	6
Jongsareejit A,2013	*0*	0	*0*	1	1	1	1	0	1	5
Mart ń-Merino E,2016	1	1	0	0	1	1	1	1	1	7
Giuffr èG, 2004	1	1	0	1	1	1	0	1	0	6
Tseng ST, 2015	1	1	1	1	1	1	0	0	0	6

(A) Define the study design, (B) List inclusion and exclusion criteria for all the participants; (C) Indicate study period and follow-up duration; (D) Diagnosis of DR was based on fundus examination or Fundus photography; (E) Provided enrollment duration for all the participants; (F) Described the general characteristics, such as age and sex, in all the participant population; (G) Adjusted confusing factors, such as age, gender, DM duration, cardiovascular factors; (H) Stratified alcohol intake into more than three stratifications; (I) If the common influence factors, including age, gender, DM duration, were matched among all the groups. A: case control or cohort study, 1 point and cross-sectional study, 0 point. The rest: Yes, 1 point and No, 0 point.
